# Duration of Antifungal Therapy in Disseminated Coccidioidomycosis Patients: A Real-World Treatment Utilization Study

**DOI:** 10.3390/jof12040293

**Published:** 2026-04-20

**Authors:** Craig I. Coleman, Belinda Lovelace, Mark Bresnik

**Affiliations:** 1 Department of Pharmacy Practice, University of Connecticut School of Pharmacy, Storrs, CT 06269-3092, USA; 2 Health Economics and Outcomes Research, F2G, Inc., Princeton, NJ 08540, USA; belinda.lovelace@f2g.com; 3 Clinical Research and Development, F2G, Inc., Princeton, NJ 08540, USA; mbresnik@f2g.com

**Keywords:** antifungal therapy, coccidioidomycosis, medication discontinuation, medication persistence

## Abstract

**Background:** Disseminated coccidioidomycosis (DCM) often requires prolonged antifungal therapy (AFT). Real-world data on AFT duration in DCM are limited. We evaluated time to AFT discontinuation among patients with DCM in the United States clinical practice. **Methods:** This retrospective, longitudinal study used STATinMED data (2016–2024). Patients had ≥1 International Classification of Diseases, Tenth Revision (ICD-10) code for DCM (B38.3, B38.4, B38.7, B38.81) during January 2017–December 2023, ≥1 claim for a triazole or amphotericin B within 21 days of the DCM diagnosis (index date), and continuous medical/pharmacy coverage during the 6-month baseline period. Discontinuation was defined as a ≥21-day gap without AFT. Antifungal agent/formulation switches were not considered discontinuations unless accompanied by a qualifying gap. The Kaplan–Meier methods were used to estimate time to discontinuation. **Results:** We identified 991 patients with DCM. Median age was 52 years (IQR 36, 65); 60.0% were men. Most resided in California (42.8%) or Arizona (33.6%). Initial AFT consisted predominantly of triazoles (96.8%), primarily fluconazole (83.2%). Discontinuation occurred in 27.6%, 40.0%, 54.2%, and 68.0% of patients by 3, 6, 12, and 36 months. Median AFT duration was 9.9 months. **Conclusions:** In a large US claims cohort, there was substantial variability in AFT duration in routine practice. Many patients had AFT durations under the lower limit of guideline recommendations for DCM, suggesting potential under-treatment, though appropriate clinical justifications may have existed.

## 1. Introduction

Coccidioidomycosis is a fungal infection endemic to the southwestern United States (US), as well as parts of Mexico and Central and South America [1,2]. Disseminated coccidioidomycosis (DCM) occurs when *Coccidioides* infection extends beyond the lungs to sites such as the skin, bones, joints, or central nervous system (CNS) [1,2]. This is estimated to occur in approximately 1% of coccidioidomycosis cases, with an estimated burden of DCM in the US as high as 3600 cases per year.

Guidance for antifungal therapy (AFT) duration in coccidioidomycosis is mostly based upon expert consensus, supported by limited controlled trial data and observational studies demonstrating high relapse rates (dependent on disease site and severity) after stopping therapy. DCM management typically requires prolonged and sometimes lifelong AFT [2]. However, the utilization of AFT for DCM in routine US clinical practice has not been thoroughly assessed. We therefore evaluated time to AFT discontinuation among patients with DCM as part of a real-world treatment utilization study.

## 2. Methods

This was a retrospective, longitudinal, observational study using the STATinMED (Dallas, TX, USA) Real-World Data (RWD) Insights all-payer claims database from January 2016 to December 2024. The dataset represents patients across the US and includes long-term medical and pharmacy claims from commercial insurance, Medicare, and Medicaid. It covers about 80% of all healthcare claims, representing roughly 300 million individuals [3]. The data include patient demographics, diagnoses, procedures, prescription fills, and healthcare use, allowing us to examine how AFTs are used and how long patients remain on treatment for DCM.

Patients were included if they had at least one International Classification of Diseases, Tenth Revision (ICD-10) diagnosis code for DCM (B38.3, B38.4, B38.7, B38.81) [4] during the identification period (January 2017 to December 2023). Patients with “unspecified” coccidioidomycosis (B38.9) (and no DCM code) were excluded because they could not be definitively classified as having or not having DCM. Patients also needed to have at least one claim for a triazole or amphotericin B within 21 days of the DCM diagnosis (index date) and continuous medical and pharmacy coverage during the 6 months preceding the index date (baseline period).

The outcome of interest for this study was AFT discontinuation, defined as a gap of at least 21 consecutive days without AFT. Switching between intravenous and oral formulations or between triazoles and polyenes did not constitute discontinuation unless a ≥21-day gap occurred. The patients were followed from the index date until the earliest of AFT discontinuation, death, loss to follow-up, or the end of the study period (December 2024) (follow-up period).

Continuous variables were summarized using medians and interquartile ranges (IQRs), and categorical variables were presented as counts and percentages. Kaplan–Meier methods were used to estimate and visualize time to AFT discontinuation over the follow-up period.

The STATinMED RWD Insights all-payer claims database is fully compliant with the Health Insurance Portability and Accountability Act of 1996 (HIPAA) [3]. As this study used only de-identified patient-level data and did not involve the collection, use, or transfer of individually identifiable information, institutional review board (IRB) approval was not required.

This study report was written in compliance with the STrengthening the Reporting of OBservational studies in Epidemiology (STROBE) statement [5].

## 3. Results

A total of 8674 patients with any ICD-10 diagnosis code for coccidioidomycosis, at least one AFT claim within 21 days before or after the initial coccidioidomycosis diagnosis, and continuous medical and pharmacy coverage during the 6-month baseline period were identified. Of these, 991 (11.4%) had a diagnosis code consistent with DCM. The median age was 52 years (IQR 36, 65), and 60.0% of the patients were men. Most patients were identified as residing in California (42.8%) or Arizona (33.6%), with 8.6% residing in low-endemic states (Table 1). CNS involvement was present in 30.6% of patients, cutaneous disease in 17.6%, and prostatic disease in 1.3%, with the remainder (50.6%) of patients having been classified broadly as disseminated disease. Concomitant pulmonary coccidioidomycosis occurred in 22.4% of the patients. Medicaid was the most common payer (38.5%), followed by commercial insurance (29.4%) and Medicare (23.1%).

**Figure 1:**
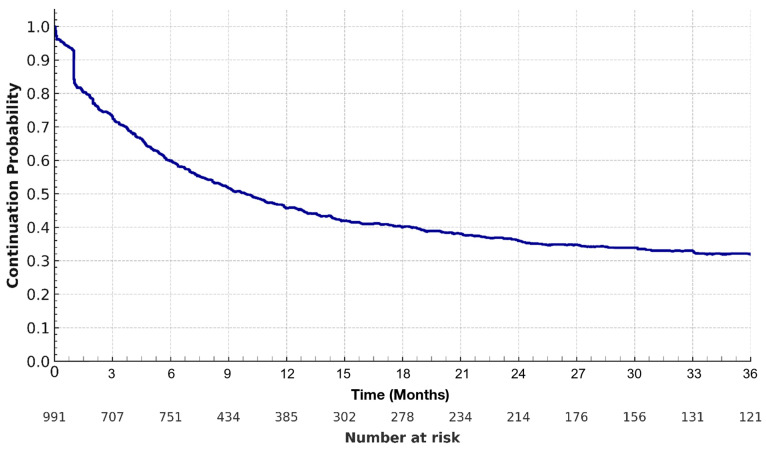
**Kaplan–Meier curve for discontinuation of antifungal therapy in patients with disseminated coccidioidomycosis**.

Triazoles were used as the initial AFT in 96.8% of the patients—primarily fluconazole (83.2%), followed by itraconazole (5.5%), voriconazole (4.9%), posaconazole (2.2%), and isavuconazole (1.4%). Amphotericin B was initially used in 3.7% of patients and in 4.3% of patients at any time after the index date. The discontinuation of AFT occurred in 27.6%, 40.0%, 54.2%, and 68.0% of patients by 3, 6, 12, and 36 months, respectively. The median AFT duration was 9.9 months (Figure 1).

## 4. Discussion

According to the 2016 Infectious Diseases Society of America (IDSA) guidelines [2], the treatment of DCM depends on the site and severity of disease. Non-CNS infection involving skin, soft tissue, or bone is managed with oral azoles such as fluconazole, while amphotericin B is reserved for severe or rapidly progressive cases. Therapy is typically prolonged, at least 6–12 months, and may extend to 2–3 years or longer for osseous or extensive soft tissue disease, with some patients requiring chronic suppressive therapy. We found that at least one-quarter of patients discontinued AFT prior to 3 months, and 40% received therapy durations lower than the lower limit of guideline recommendations for DCM (<6 months), potentially pointing to under-treatment of DCM in some patients. However, our inability to split patients into reliable DCM manifestation types should be considered when interpreting these data. We also showed that nearly one-half (45.8%) of patients received AFT for >12 months. The median AFT duration was 9.9 months; however, this result is likely skewed toward a longer AFT duration due to the large proportion of coccidioidal meningitis patients in our population.

For coccidioidal meningitis, fluconazole is recommended because of reliable cerebrospinal fluid (CSF) penetration, and lifelong therapy is advised due to relapse risk; intrathecal amphotericin B is reserved for refractory cases. The predominance of fluconazole use in our study aligns with the above recommendations and is consistent with a prior study by Edwards and colleagues which found that 87.4% of all coccidioidomycosis cases initiated therapy with fluconazole, though a larger percentage of DCM patients started amphotericin B in lieu of a triazole [6]. While the study by Edwards et al. provided important data on coccidioidomycosis treatment pathways, including AFT switching, they did not report on the duration on AFT in their population.

We used the data from an all-payer claims database to conduct this study. The claims data are the standard source for assessing refill-based medication discontinuation because they provide objective, time-stamped dispensing records (fill dates and days’ supply) that enable reproducible definitions of discontinuation and refill gaps [7]. In this study, claims-based persistence captured continued medication availability rather than confirmed ingestion (adherence). Therefore, if some patients filled prescriptions but did not take them as prescribed, persistence may have been overestimated. Conversely, if some AFT fills occurred outside data sources captured in the STATinMED dataset (e.g., cash payments, samples, other insurers, or non-captured pharmacies), persistence may have been underestimated.

This study has several additional limitations. The potential for misclassification bias exists due to our reliance on billing codes to identify patients with DCM [8]. While it is reasonable to assume patients with an ICD-10 diagnosis code for DCM in a claims data set in fact have the disease, it is also possible that there is under-ascertainment (missed cases) of DCM. This may particularly be the case when “unspecified” coccidioidomycosis ICD-10 diagnosis codes (B38.9) are used by clinicians. Prior literature suggests that anywhere between 17.8 and 49.5% of coccidioidomycosis cases may be coded as “unspecified” disease, and about two-thirds (67.9%) of patients with an “unspecified” code could have been assigned a more specific coccidioidomycosis code, including 38.9% that could have been assigned a DCM code [9]. Notably, our case identification followed the same ICD-10 schema used in prior Centers for Disease Control and Prevention (CDC) analyses of coccidioidomycosis [4]. While specific ICD-10 codes for coccidioidal meningitis (B38.4), as well as cutaneous (B38.3) and prostatic (B38.81) disease, exist, codes distinguishing other disseminated sites (e.g., bone, joint) or severity of disease (e.g., rapidly progressive disease, extensive soft-tissue involvement) are lacking. Moreover, a more generic DCM code is available (B38.7), and it is commonly used. For these reasons, we were unable to evaluate the association between the AFT duration and clinical outcomes, as we could not account for individual patient disease severity. Finally, the findings of our population drawn only from the STATinMED RWD Insights all-payer claims database may not be generalizable to the entire DCM population in the US.

## 5. Conclusions

Triazoles were initiated in nearly all identified DCM patients. Forty percent of patients received AFT durations lower than the lower limit of guideline recommendations (<6 months) for DCM treatment, which may suggest too short of a treatment duration for DCM in some patients, though caution should be exercised when drawing too sharp a conclusion, given the limited data on specific DCM manifestations. Nearly one-half of patients received treatment for longer than 1 year. As new antifungals for coccidioidomycosis enter practice (e.g., fosmanogepix, olorofim), real-world data on how long patients remain on therapy will be essential for payers and providers to forecast resource use and to design access policies that match expected treatment courses. Future studies should examine clinical, economic, and behavioral factors contributing to AFT cessation by disease manifestation that could help identify potential interventions and improve long-term treatment persistence in DCM.

## Figures and Tables

**Table 1:** Characteristics of included disseminated coccidioidomycosis patients.

	**DCM** **(N = 991)** **n (%)**
Index Year	
2017	140 (14.1)
2018	178 (18.0)
2019	147 (14.8)
2020	154 (15.5)
2021	129 (13.0)
2022	124 (12.5)
2023	119 (12.0)
Age, Years, Median (IQR)	52 (36, 65)
Male Sex	595 (60.0)
US Geographic Region	
California	424 (42.8)
Arizona	333 (33.6)
Low Endemicity *	85 (8.6)
Unknown Endemicity ^†^	149 (15.0)
Payer	
Medicare	228 (23.1)
Medicaid	381 (38.5)
Commercial	291 (29.4)
Other	89 (9.0)
Unknown	2 (0.2)
Coccidioidomycosis Location	
Central Nervous System	303 (30.6)
Pulmonary	222 (22.4)
Charlson Comorbidity Index, Median Score (IQR)	1 (0, 3)
0	449 (45.3)
1	195 (19.7)
2	61 (6.2)
3	60 (6.1)
4	30 (3.0)
5+	196 (19.8)
Baseline Comorbidities	
COVID-19	32 (3.2)
Pneumonia	361 (36.4)
Chronic pulmonary disease	163 (16.5)
Any malignancy (except neoplasm of skin)	106 (10.7)
Hematologic malignancies	39 (3.9)
Transplant history or complications	28 (2.8)
AIDS/HIV	49 (4.9)
Congenital or acquired immunodeficiencies	63 (6.4)
Autoimmune diseases	59 (6.0)
Connective tissue/rheumatic disease	40 (4.0)
Recent/chronic systemic corticosteroid use	147 (14.8)
Diabetes	182 (18.4)
Obesity	128 (12.9)
Renal disease	107 (10.8)
Moderate-severe liver disease	110 (11.1)
Myocardial infarction	34 (3.4)
Heart failure	72 (7.3)
Peripheral vascular disease	51 (5.2)
Cerebrovascular disease	89 (9.0)

AIDS = acquired immunodeficiency syndrome; DCM = disseminated coccidioidomycoses; HIV = human immunodeficiency virus; IQR = interquartile range; US = United States. * Low endemicity regions for coccidioidomycosis in the US include Nevada, New Mexico, Texas, Utah, and Washington. ^†^ Unknown endemicity refers to all states not captured in low endemicity, Arizona, or California.

## Data Availability

The data utilized in this study are available only by license through STATinMED.
